# Clinical and Neurobiological Perspectives of Empowering Pediatric Cancer Patients Using Videogames

**DOI:** 10.1089/g4h.2015.0014

**Published:** 2015-10-01

**Authors:** Meveshni Govender, Randy C. Bowen, Massiell L. German, Grzegorz Bulaj, Carol S. Bruggers

**Affiliations:** ^1^ Division of Hematology-Oncology, University of Utah School of Medicine, Salt Lake City, Utah.; ^2^ Department of Pediatrics, University of Utah School of Medicine, Salt Lake City, Utah.; ^3^ Department of Medicinal Chemistry, College of Pharmacy, University of Utah, Salt Lake City, Utah.; ^4^ Huntsman Cancer Institute, University of Utah School of Medicine, Salt Lake City, Utah.; ^5^ Primary Children's Hospital, Salt Lake City, Utah.

## Abstract

Pediatric oncology patients often experience fatigue and physical and mental deconditioning during and following chemotherapy treatments, contributing to diminished quality of life. Patient empowerment is a core principle of patient-centered care and reflects one's ability to positively affect his or her own health behavior and health status. Empowerment interventions may enhance patients' internal locus of control, resilience, coping skills, and self-management of symptoms related to disease and therapy. Clinical and technological advancements in therapeutic videogames and mobile medical applications (mobile health) can facilitate delivery of the empowerment interventions for medical purposes. This review summarizes clinical strategies for empowering pediatric cancer patients, as well as their relationship with developing a “fighting spirit” in physical and mental health. To better understand physiological aspects of empowerment and to elucidate videogame-based intervention strategies, brain neuronal circuits and neurotransmitters during stress, fear, and resilience are also discussed. Neuroimaging studies point to the role of the reward system pathways in resilience and empowerment in patients. Taken together, videogames and mobile health applications open translational research opportunities to develop and deliver empowerment interventions to pediatric cancer patients and also to those with other chronic diseases.

## Introduction

Empowerment is a core process of patient-centered care that promotes patient engagement in and accelerates improvements of mental and physical health. Empowerment reflects the ability of patients to actively understand and positively influence their own health status.^1–4^ It involves connections and support from healthcare providers, family, and friends to restore a personal sense of hope, respect, and self-efficacy.^4–6^ Empowerment is a multidimensional concept involving access to resources and the development of a “fighting spirit”—the enduring drive to refuse to surrender to seemingly insurmountable challenges.^7^ In this article, we review the empowerment interventions for pediatric oncology patients, while emphasizing clinical and translational research opportunities for serious videogames in this new area of medicine.

## Empowerment Interventions in Pediatric Oncology

Although childhood cancer-directed medical interventions have been extensively studied, children's psychological experiences and quality of life (QOL) associated with childhood cancer have received comparatively limited attention. Research aimed at understanding and improving psychosocial outcome of children with cancer and their families is essential. Children diagnosed with cancer are significantly more likely to experience compromised mental health and QOL compared with siblings and peers.^8^ Omnipresent patient and parent stresses include prolonged hospitalization, altered body image and sense of vitality, and fear of death. Treatment usually involves surgery, chemotherapy, and radiation therapy and is oftentimes arduous, invasive, and lengthy. Related stress experiences involve the actual event, the child's making sense of the event, the search for coping strategies, both behavioral and cognitive, and finally implementation of these strategies.^9,10^ Adolescent cancer patients may feel a sense of inadequacy, loss of control, and frustration, resulting in compliance issues.^11^

Optimal adjustment to such uncontrollable stressors often requires adjusting oneself to the stressors rather than one being able to change the stressors (i.e., adjusting one's perceptions and reactions to a situation when one cannot alter the situation). Children with a wide range of coping strategies feel more in control and thus experience less anxiety than those with a narrow range of coping strategies.^8,12,13^ Current strategies addressing these comorbidities include counseling, physical therapy, and pharmacological therapies, alone or in combination.^14–19^ A key feature shared by all of these approaches is that they are externally administered to the individual.

**FIG. 1.**
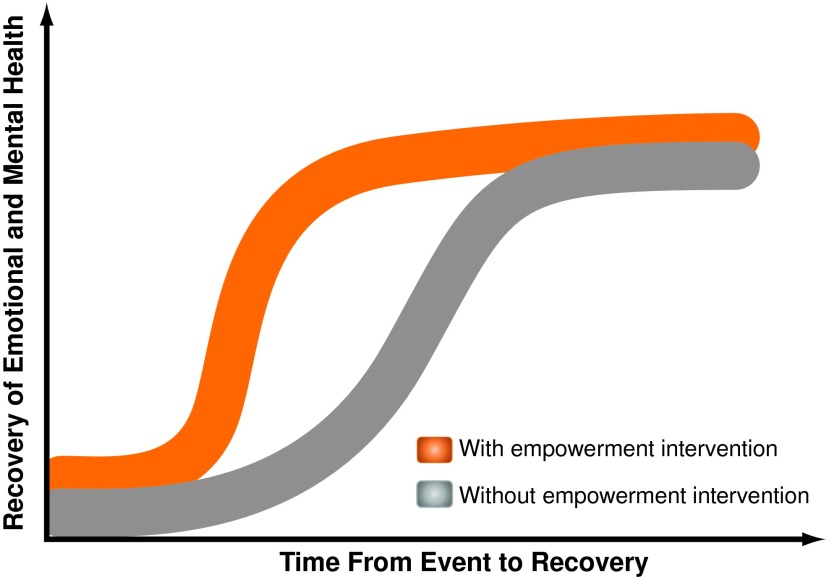
Empowerment accelerated improvements in physical and mental health of a patient, as shown by two possible recovery trajectories. Effectiveness of patient empowerment can be monitored by time dependence of health-related quality of life metric tools. (Color graphics available at www.liebertonline.com/g4h)

A complementary approach to addressing patient depression, fear, generalized deconditioning, and fatigue involves the concept of empowerment—an enhanced mental ability to understand and positively influence one's own health status by patient engagement in his or her health management.^1–6^ As illustrated in Figure 1, empowerment can be defined in clinical terms as a process that accelerates and enhances improvements of both mental and physical health. Current empowerment-centered interventions include printed documents, conferences for cancer survivors, cancer camps, group sessions, and exercise programs (Table 1). Addressing patient and parental concerns with one-on-one education regarding diagnosis, treatment plan, and potential side effects, as well as providing emotional and social support directed at increasing confidence, can reduce anxiety and help patients feel in control of their health situation.^14–16^ Beneficial results of empowerment-centered interventions have already been demonstrated in patients with diabetes and attention deficit hyperactivity disorder.^26–29^

**Table 1.** Studies Illustrating Benefits of Interventions Directed at Children and Adolescents with Cancer

*Intervention*	*Description*	*Outcome measures*	*Benefits*	*References*
Oncology camps	Therapeutic or recreational programs designed for children with cancer alone or together with their parents and/or siblings, usually in an environment that is controlled, safe, and away from stressors of home/hospital. Attendees participate in activities with the goal of improving physical, psychological, or social functioning.	Diverse measures, detailed in respective references	Improved cancer knowledge through peer learning; increased feelings of autonomy and self-confidence; improved mood and quality of life; improved friendship skills and relationships with family members; improved coping and functioning at home	Bradlyn et al.,^14^ Martiniuk et al.,^17,18^ Bluebond-Langner et al.^19^
Group interventions: Opkoers	Opkoers is a standardized group intervention deigned to empower children with diverse chronic illnesses, including cancer, using cognitive behavioral techniques. Children 8–18 years old attend six sessions in which techniques designed to improve the following areas are taught: information seeking and giving; positive thinking; and relaxation and social competence.	Opkoers Questionnaire for parent and child; Cognitive Control Strategies Scale to determine outcomes of intervention; Dutch Child Behavior Checklist; State Trait Inventory for Children; Self Perception Profile for Children and Adolescents; DUX25 assessment of social and emotional outcomes	Short- and medium-term improvements in information seeking, positive thinking, and social competencies; fewer reported behavioral and emotional problems by parents; improved quality of daily functioning	Last et al.,^20^ Scholten et al.^21^
Group intervention: Opkoers Onkolgie	The above-described Opkoers intervention was adapted to include cancer-specific elements aimed at pediatric cancer survivors. A pilot study (*n*=11) was conducted to determine appropriateness for future use.	Opkoers Questionnaire for parent and child; Cognitive Control Strategy Scale to determine outcomes of intervention	Improvements in social competencies and positive thinking were seen, and the intervention was deemed appropriate for adolescent cancer survivors. A cancer-specific module has been successfully implemented, with an adapted computer-accessible version created.	Maurice-Stam et al.^22^
“I'm Cured…Now What? A Conference for Teen and Young Adult Survivors of Childhood Cancer” (annual conferences 2006–2012)	Multidisciplinary day-long conference for childhood cancer survivors >17 years of age, including lectures on diverse topics such as survivorship, introduction to new therapies, small group sessions, and networking opportunities, as well as an incentive to improve conference attendance	Retrospective survey of survivor attendees of four different programs (*n*=65)	Increased knowledge regarding late effects of therapy and the need for healthy diet, physical exercise, and stress reduction	Sadak et al.^23^
Exercise intervention	Structured exercise programs given at home, sporting centers, or in hospital setting that included endurance, strength, aerobic, and/or coordination exercises	Measurements of feasibility, adverse effects, quality of life, and fatigue were used.	Majority of interventions were feasible. Improved quality of life and decreased cancer-related fatigue were shown. No adverse effects were reported.	Baumann et al.,^24^ Chang et al.^25^

It is interesting that although interventions directed at increasing patient empowerment in diverse healthcare settings are becoming more prevalent, designing meaningful metric tools to specifically assess intervention efficacy remains challenging. Such interventions were initially developed for use in mental health populations and more recently expanded to assess other patient populations, including those with diabetes, cancer, and other chronic diseases, as described in Table 2. Although some of these studies have used validated tools, other studies have used simple, brief, nonvalidated questionnaires aimed at determining intervention efficacy.

**Table 2.** Metric Tools Designed to Assess Empowerment

*Study aim*	*Methods/population*	*Metric tool*	*Outcome*	*Reference*
General purpose, condition-specific tool designed to measure locus of control	588 patients with rheumatoid arthritis, chronic pain, diabetes, or cancer	18-item general purpose, condition-specific locus of control scale adaptable for diverse medical or health-related conditions	Reliability and validity of the four Form C subscales—Internality, Chance, Doctors, and Other (powerful) People—were demonstrated.	Wallston et al.^30^
To define a scale to measure the personal construct of empowerment	271 members of six self-help programs in several states	Consumer-constructed scale to measure empowerment among mental healthcare users	Identified five factors: self-esteem; power–powerlessness; community activism; righteous anger; optimism—control over the future	Rogers et al.^31^
To validate a specific tool in adult outpatient mental health population	Participants in a state outpatient mental healthcare system	Empowerment Scale	Confirmation of five subscales of empowerment: esteem; power; activism; anger; and control	Wowra and McCarter^32^
To determine construct validity of empowerment among consumers of mental healthcare services	Users of mental health care services	Empowerment Scale	Self-empowerment as associated with quality of life, social support, self-esteem, and psychiatric symptoms. Community empowerment was correlated with self-esteem, resources, verbal intelligence, and ethnicity.	Corrigan et al.^33^
To develop and validate a questionnaire measuring empowerment in personal health care and services	873 participants in program of Research to Integrate Services for Maintenance of Autonomy	HCEQ	Confirmation of multidimensional concept of empowerment	Gagnon et al.^34^
To measure one's confidence in ability to carry out specific goal-directed behavior	Participants in a videogame designed to improve behavioral outcomes in young cancer patients	Cancer-specific Self-efficacy Scale developed for this study	Using this tool, significantly improved self-efficacy was demonstrated in the intervention group over time compared with baseline.	Kato et al.^35^
To measure cancer knowledge as result of specific intervention	Participants in a videogame designed to improve behavioral outcomes in young cancer patients	18-item multiple choice questionnaire specifically designed for this study to measure patients' cancer knowledge delivered in a videogame	Using this tool, significantly improved cancer-related knowledge was demonstrated in the intervention group over time compared with baseline.	Kato et al.^35^
To describe and assess available tools measuring disability that could be used in developing countries	Systematic comprehensive literature review	17 different empowerment metric tools identified	Empowerment Scale is the most widely used in developing countries but further validation indicated	Bakker et al.^36^
To systematically review literature regarding interactive Web-based empowerment interventions in cancer and chronic disease	PubMed, Embase, and Scopus database searches	Diabetes Empowerment Scale; Heart Failure Self-care Behavior Scale; Likert scale; Patient Activation Measure Self report; single question on a 1–10 scale; Cancer Behavior Inventory; Kansas City Cardiomyopathy Questionnaire; Perceived Competence Scales	Seven common elements applicable to electronic health to improve cancer survivors' empowerment: education; self-monitoring; tailored feedback; self-management symptom management training; personalized exercise program; communication with healthcare providers; and communication with fellow patients	Kuijpers et al.^37^
To operationalize cancer empowerment as an outcome measure by describing psychometric properties of an empowerment questionnaire in breast cancer survivors	140 breast cancer survivors	40-item CEQ	CEQ can measure individual patient cancer empowerment by encompassing interpersonal and intrapersonal aspects.	van den Berg et al.^38^
To investigate reliability and validity of a questionnaire designed to measure empowerment	388 male and female patients with type 2 diabetes mellitus	Self-completed, concise empowerment questionnaire	Questionnaire showed internal consistency, construct validity, reproducibility, factorial construct validity, and concurrent validity in the targeted patient population.	Hara et al.^39^
To validate scales from the heiQ	Questionnaire completed by 731 adults treated for recent cancer. Validity and reliability were then assessed.	heiQ	Validity of heiQ scales as generic measures of cancer health-related empowerment was supported.	Maunsell et al.^40^
To determine if SCM improved emotional well-being, empowerment, and symptom prevalence during chemotherapy	97 adult participants with diverse cancers were randomized to SCM or standard of care. Assessment was done before, during, and after chemotherapy.	Hospital Anxiety and Depression Scale, the Mimi-Mental Adjustment to Cancer, and Patient Empowerment Scale	Significant decrease in clinical anxiety in treatment group. No significant change in empowerment, symptom prevalence, or Mini-Mental Adjustment to Cancer and unchanged depression over time in the control group	Johnson et al.^41^

CEQ, Cancer Empowerment Questionnaire; HCEQ, Health Care Empowerment Questionnaire; heiQ, Health Education Impact Questionnaire' SCM, Shared Care Model.

## Advances in Videogame Technology and Mobile Health Applications

With the advent of computers and multimedia, attention has been drawn to alternative sources of education and empowerment. Computer-based interventions in the form of compact disk read-only memory, Web/Internet-based interventions, and videogames, as detailed in Table 3, are increasingly being studied. These patient-centered, interactive modalities have a greater appeal to children and adults.^42–49^

**Table 3.** Studies Illustrating Benefits of Computer-Based Interventions in Disease Management

*Study aim*	*Outcome*	*References*
Evaluation of an interactive CD-ROM versus a book about leukemia in children with leukemia	Compared with the book, (1) children using the CD-ROM showed and increased feeling of control over their health, and (2) children and parents were more satisfied using the CD-ROM and used it more frequently and for longer time periods.	Dragone et al.^42^
Evaluation of an interactive CD-ROM designed for adolescents with cancer versus a handbook	Compared with the handbook, there were greater acceptability of the CD-ROM and increased internal locus of control in teens.	Jones et al.^44^
To determine whether participation in online support groups has an effect on patient empowerment in adults with chronic diseases	Patients reported improvements in knowledge, social contact, and feelings of confidence, optimism, and control and felt more prepared to interact with healthcare professionals.	van Uden-Kraan et al.^45^
To determine the effect of an online cognitive behavioral program (MoodGYM) designed to decrease and prevent symptoms of depression and anxiety in adolescents	Decreased anxiety symptoms in both males and females and decreased depression symptoms in males were found.	Calear et al.^46^
To determine effect of playing a virtual reality game on procedural pain in children with acute burn injuries	Virtual reality coupled with analgesics was significantly more efficacious in pain reduction than analgesics alone.	Das et al.^47^
To examine feasibility and safety of establishing a virtual community in children undergoing chronic hemodialysis in a hospital setting	“Zora” was enjoyable and safe and provided pediatric renal patients with a means of coping with chronic physical illness.	Bers et al.^48^
To examine feasibility and safety of using a Web-based virtual community for psychosocial support in adolescents in the home setting following solid organ transplantation	“Zora” was safe and effective as a potential psychosocial intervention for adolescents at home following solid organ transplantation.	Bers et al.^49^

Designing interactive videogames specifically for pediatric oncology patients is an exciting area of interest. A study involving virtual reality computer games on depressive symptoms in Hong Kong Chinese hospitalized children with cancer was recently conducted. Patients in the study group, which included videogame play therapy, had statistically significant fewer depressive symptom than those in the control group.^50^ “Re-Mission™” is a sedentary interactive videogame created to change behavior and improve treatment medication adherence of children and young adults diagnosed with acute lymphoblastic leukemia.^35,51^ In a multicenter randomized control trial involving 375 patients 13–29 years of age, patients whose treatment included playing “Re-Mission” as well as standard therapy (treatment group) showed a statistically significant improvement in adherence to prescribed medication regimens compared with patients treated with standard therapy alone, despite suboptimal use of the intervention by most participants.^35^ Playing “Re-Mission” also resulted in a significantly greater increase in cancer-related knowledge when compared with the control group, likely resulting from information integrated within the game or information-seeking behavior from the videogame therapy.^51^ These studies provide important evidence that a judiciously designed videogame can enhance certain behavioral outcomes, potentially promote information seeking, and increase knowledge in adolescents and young adults with cancer.

Adapting existing commercially available videogames, including “Dance Dance Revolution” (Konami Digital Entertainment, El Segundo, CA), “Wii™ Bowling” and “Wii Boxing” (Nintendo, Kyoto, Japan), and “Circus Challenge” (Limbs Alive, Newcastle upon Tyne, United Kingdom), to promote physical exercise and weight loss and to improve neurologic function, has also become an important therapeutic approach.^52–57^ However, videogames specifically designed for particular population with a specific therapeutic goal are sparse and generally sedentary in nature.

The progression of videogames and mobile applications (apps) designed specifically for therapeutic benefit has focused on education and promotion of healthy life styles via diet, as well as fitness and exercise, in adults, and few are directed at children or specific diseases. However, one example of a child-focused game is “Zora,” a three-dimensional graphical virtual multiuser community designed for children undergoing chronic hemodialysis in the hospital setting, provided a safe and novel means for coping and sharing experiences in the creation of a virtual city/community support network.^48^ “Zora” was subsequently found to be feasible, safe, and beneficial as an Internet-based psychosocial intervention in the home setting for adolescents following solid organ transplantation.^49^

In a study focusing on clinical depression, patients were randomized to either receive videogame therapy (treatment group) or no videogame therapy (control group). Using the Patient Health Questionnaire, patients in the treatment group showed a statistically significant reduction of depressive symptoms at 1 month.^58^ The same research group recently reported that playing casual videogames as a prescribed medical treatment also help in reducing anxiety.^59^

In another study, traditional face-to-face cognitive counseling therapy for depression was compared with a computerized cognitive behavior therapy (SPARX) over 7 months; a statistically significant improvement in depression rating was demonstrated in the SPARX cohort compared with the traditional treatment cohort.^60^ Commercially available videogames used as distraction therapy have also resulted in decreased conditioned nausea.^61,62^ Finally, in a randomized controlled trial involving pediatric burn patients, the average procedural pain scores, measured using the Faces Scale, were significantly less in the group where playing a virtual reality game was used in addition to traditional analgesia.^47^

Creating hope, improving QOL, increasing one's sense of control, and reducing stress are also important parts of patient empowerment, especially in pediatric oncology. With these concepts in mind, the prototype “Patient Empowerment Exercise Videogame” (the PE Game) was developed.^63^ The PE Game is an interactive videogame specifically designed for pediatric oncology patients during their chemotherapy treatment.^63^ This videogame couples physical exercise with patient empowerment through several minigames that visualize metaphors for fighting cancer and provide positive reinforcement via physical activity. The nonviolent themes encourage safe physical exercise while avoiding repetitive stress and violence for the patient, who assumes the role of a superhero overcoming diverse obstacles.^63,64^ Patient- empowering videogames, such as the PE Game, offer a personalized support and improvement of disease self-management for patients of all ages with diverse chronic conditions.^65^

Mobile health (m-health) is a rapid expansion from the growing electronic healthcare practices. Between 2002 and 2012 approximately 117 articles were published on the impact of mobile phones and smartphones in health care.^66^ With the expansion of smartphone systems and cellular network technologies, novel biomedical sensors and communication paths continue to advance, creating room for m-health development.^67^ The use of these devices in low- and middle-income countries is also of interest for healthcare maintenance and telemedicine.^68^ Some smartphone applications promote healthier lifestyles and dietary choices, mental and behavioral health, obesity prevention, exercise monitoring, and blood glucose data analysis.^69–71^

The importance of mobile interventions is illustrated by development of “Re-Mission 2,” a mobile online game of Re-Mission, that promotes empowerment, self-efficacy and positive emotions, while also motivating pediatric and adolescent cancer patients to medication adherence. Another example is the U.S. Food and Drug Administration–approved BlueStar^®^ (WellDoc, Baltimore, MD) mobile platform used for behavioral self-management in diabetic patients.^72,73^ For empowerment interventions, there are already available mobile apps for stress and anxiety management, including gamified mobile apps specifically focused on cognitive training or biofeedback-coupled music apps.^74–76^ Given increasing popularity of biofeedback-based wearables, mobile apps can bridge virtual and real worlds to empower patients.^59,77^ With clinical development and validation, therapeutic videogames and m-health have great promise of becoming personalized medicine tools in health care.^78^

## Neurological Mechanisms in Emotional Homeostasis

**FIG. 3.**
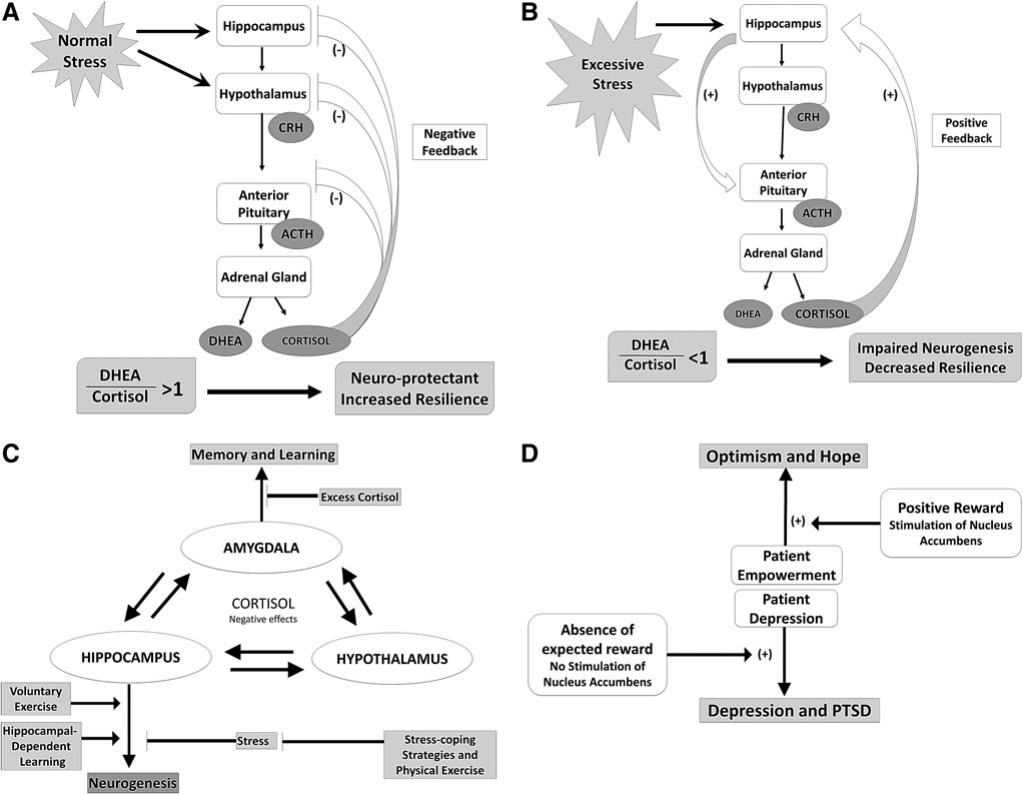
Overview of relationships among brain structures, hormone feedback systems, and psychosocial conditions following stress exposure. **(A)** A healthy response following stress results in a favorable dehydroepiandrosterone (DHEA):cortisol ratio of greater than 1, as a result of negative feedback inhibition of cortisol on the hypothalamic–pituitary–adrenal axis. Such a condition is neuroprotective and may promote resilience. ACTH, adrenocorticoptropic hormone; CRH, corticotrophin-releasing hormone. **(B)** Following excessive stress, failure of this negative feedback can result in continued cortisol production and hence an unfavorable DHEA:cortisol ratio of less than 1. This may result in impaired neurogenesis and decreased resilience. **(C)** Physical exercise and stress-coping strategies can decrease stress and thus promote neurogenesis, learning, and improved memory. **(D)** Stimulation of the nucleus accumbens by positive rewards enhances patient empowerment and promotes optimism and hope. In contrast, absence of an expected reward results in no stimulation of the nucleus accumbens and can increase depression and/or posttraumatic stress disorder (PTSD).

Emotional homeostasis and empowerment are intrinsically interconnected within an individual's neurobiology (Fig. 3). An intact reward system is central for preserving optimism and hope, qualities identified in empowered individuals, when confronted with adversity in everyday life, whether mild or extreme.^79^ Two behavioral systems integral in normal human behavior include one motivating the pursuit of potential rewards and one motivating avoidance of potential losses.^79^ The neural circuitries of stress, fear, and the mesolimbic dopamine pathway, also known as the reward system, are overlapping and interrelated in adaptive responses to fear and stress and in building resilience and empowerment.^80–85^ The predominant neurotransmitter of the reward system is dopamine, sometimes referred to as the pleasure chemical of the brain. Common brain regions with reciprocal interconnections include the hypothalamus, hippocampus, amygdala, prefrontal cortex, orbital frontal cortex, nucleus accumbens, the hippocampus, and the amygdala.^86,87^ The hippocampus and amygdala are central in emotional memory encoding and consolidation and in generating and regulating emotional reactions in response to stress, regulated in part by cortisol.^86–89^

The medial prefrontal cortex and the orbital frontal cortex are central in reward learning and guiding future behavior based on anticipated rewards or punishments.^90–92^ The orbital frontal cortex receives direct input from the taste, olfactory, visual, and somatosensory areas and is involved in integration of multiple stimuli and value assessment of stimuli.^90,91^ The medial prefrontal cortex is associated with visceral function in relation to emotion.^93^ Lesions in these areas result in an inability to make reward-based decisions and to distinguish rewards from punishments. The inability to predict future rewards has been implicated in depression because it is thought to result in a hopeless and pessimistic outlook.^91^ Decreased dopamine signaling, with resultant diminished striatal activation, has been documented in and associated with symptomatic depression and posttraumatic stress disorder in patients.^79,81,82^

Hormones important in maintaining emotional homeostasis in times of fear and stress include (1) corticotrophin-releasing hormone, which is anxiogenic when stimulating corticotrophin-releasing hormone 1 receptors but anxiolytic when stimulating corticotrophin-releasing hormone 2 receptors, (2) cortisol, which is important in restoration of homeostasis following stress, (3) neuropeptide Y, which is endogenously anxiolytic, and (4) dehydroepiandrosterone (DHEA), with neuroprotectant functions including promoting neurogenesis, establishing memory, and endorphin synthesis.^84,88,89,94–97^ The effect of neuropeptide Y as an endogenous anxiolytic has been compared with that of benzodiazepines.^85,88,91,92^ Neuropeptide Y, the level of which is known to increase with stress, was significantly increased from baseline following survival training, and chronic overexpression of neuropeptide Y has been associated with stress insensitivity.^88^ Empowerment interventions that have the ability to stimulate the reward circuit while suppressing the fear, anxiety, and stress response could play a role in both treatment and prevention of depression and posttraumatic stress disorder in cancer survivors.^80–84^

## Translational Research and Development of Empowerment Interventions

Understanding the effect that empowerment interventions have on neural systems facilitates the development of interventions that may alter the structure and functional brain changes associated with early life adversity.^85^ Maintaining a properly functioning reward system when confronted with extreme adversity is crucial to preserving optimism and hope, both of which are qualities identified in empowered individuals.^79^ Empowerment-promoting interventions targeting brain areas and circuitries described above, including interactive videogames designed to stimulate reward neural circuitry, have the potential to ameliorate the effects of chronic stress and improve resilience in patients with an increased risk of anxiety and depression, including those undergoing treatment for cancer.

One illustrative example is the above-described sedentary videogame “Re-Mission,” designed to improve disease self-knowledge and medicine compliance in teenagers with cancer. Functional magnetic resonance imaging (fMRI) was used to elucidate brain activity involved in active versus passive gameplay in healthy volunteer undergraduate students while they either played or observed someone else play “Re-Mission.” The fMRI results showed (1) active gameplay resulted in increased left parahippocampal cortex activation, (2) active gameplay, as opposed to passive observation, was necessary for activation of the mesolimbic reward system, and (3) there was a positive correlation between left parahippocampal activation and attitudes toward chemotherapy when assessed immediately following and then 1 month following gameplay.^35,51,97^

Because the hippocampus plays a role in emotional learning and memory consolidation, it is not surprising that they found a positive correlation between the degree of left parahippocampal activation and positive attitudes toward chemotherapy after playing the game and at the 1-month follow-up. Similarly, the role of enhanced reward system in building resilience has been shown in fMRI brain studies in Special Forces soldiers.^98^ Finally, in patients with treatment-resistant depression, deep brain stimulation in the nucleus accumbens has been shown to improve symptoms of depression.^81,83,99^

Taken together, these data suggest that interventions directed at stimulating the complex reward circuitry and/or suppressing fear, anxiety, and stress responses can lead to improvements of core symptoms of depression. Interventions directed at decreasing the cortisol:DHEA ratio, by either decreasing cortisol or increasing DHEA levels, could promote resilience and serve as potential targets for therapeutic intervention, including carefully designed therapeutic videogames.^84,99–101^ Measurement of the cortisol:DHEA ratio could serve as a useful indicator of efficacy of such interventions. Such interventions could be exogenously administered pharmacologic compounds or disease-directed videogames specifically designed to stimulate and/or augment the reward neural circuitry, and thus decrease or prevent depression and stress disorders, and to promote empowerment in children and others with cancer.^81–84,90,91^ Of equal importance to understanding mechanisms of empowerment interventions are advancements in diagnostic tools that allow monitoring efficacy. There are new opportunities to correlate imaging and biomarkers with questionnaires assessing patient empowerment. Such questionnaires are being developed and validated for chronically ill patients.^102,103^

In addition to translational research, development of videogames as empowerment interventions may impact medical and regulatory practices. Both patients and healthcare providers will benefit from clinically validated games and apps that improve healthy habits, self-management, and QOL of chronically ill patients. “Re-Mission” and “Re-Mission 2” illustrate these benefits for pediatric oncology patients. Given that the U.S. Food and Drug Administration has been approving games and apps for medical uses (for example, BlueStar, the Jintronix [Montreal, QC, Canada] Rehabilitation System^®^, or MusicGlove^®^ [Flint Rehabilitation Devices, Irvine, CA]), there are also new opportunities for pharmaceutical and biotech industries to integrate these digital technologies with specific drug-based treatments as innovative molecular–behavioral combination therapies.^104^

## Conclusions

Patient empowerment is a complex journey of personal change that results in gaining power over one's life. Traditionally, empowerment interventions have been externally administered, and efficacy of these interventions is determined largely by patient and provider feedback through the use of questionnaires. As therapeutic paradigms shift toward neurobiology-centered care, new interventions and outcome measures targeting specific neurocircuitry central to pleasure, reward, resilience, and empowerment can be developed, and efficacy can be measured using a combination of fMRI, neurochemical, and psychological tools.^91,97–99,105^ A well-designed videogame encompassing properties such as enhanced intrinsic motivation, active imagination, and attention to and engagement in intriguing “stories” focusing on a given health-related behavioral issue could actively capture one's full attention and thus promote health behavioral change.^106^ Judiciously developed videogaming technology and mobile health are becoming valuable conduits of recruiting specific pathways and circuitry known to be involved in “positive” addiction into development of empowerment and resilience and promote optimal self-management.^107^ Using technology to improve individual empowerment and resilience via strengthening stress and pleasure-sensitive neural processes and positive feedback of stress-buffering neurotransmitters is the next facet to developing empowerment in patients of diverse ages and disease processes.

## Figures and Tables

**FIG. 2.**
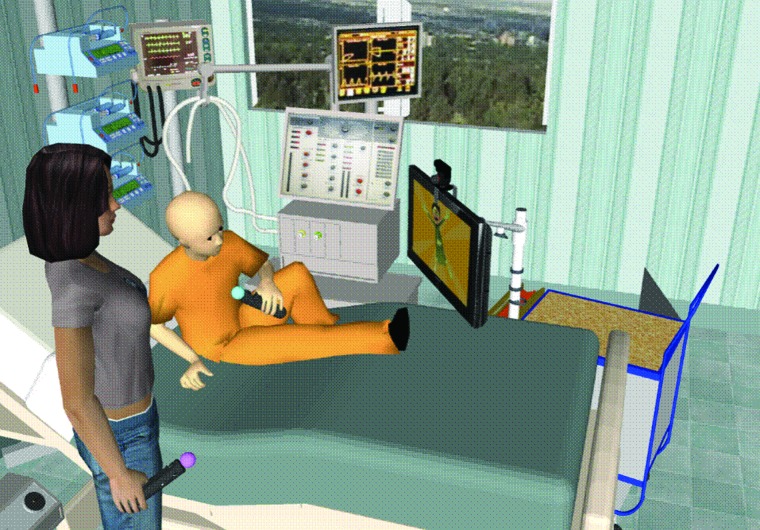
The “Patient Empowerment Exercise Videogame” being played in a hospital setting. The prototype incentive-based “Patient Empowerment Exercise Videogame” directly couples therapeutic physical exercise to mental empowerment of fighting cancer in pediatric cancer patients via visualization, positive metaphors, and active participation. It is composed of integrated, metaphoric minigames in which players, represented by friendly characters, engage in self-empowering contests on the screen, either alone or in cooperation with others (physicians, nurses, caregivers, family, friends), against the robotic “foe” avatar—cancer. Design focuses on patient safety and on avoiding violence and repetitive stress. Hand controllers sense motion speed, acceleration, and direction. Visual and audio responses provide immediate feedback and positive reinforcement. In addition to testing physiological (heart rate variability) and psychological (empowerment, locus of control) parameters, salivary levels of cortisol and neuropeptide Y are measured as surrogate biomarkers of stress and resilience. (Color graphics available at www.liebertonline.com/g4h)

## References

[1] Andersen BL Psychological interventions for cancer patients to enhance the quality of life. J Consult Clin Psychol 1992; 60:552–568 1506503 10.1037//0022-006x.60.4.552 PMC2743106

[2] Anderson RM, Funnell MM Patient empowerment: Myths and misconceptions. Patient Educ Couns 2010; 79:277–282 19682830 10.1016/j.pec.2009.07.025 PMC2879465

[3] Furnham A, Steele H Measuring locus of control. Br J Psychol 1993; 84:443–479 8298858 10.1111/j.2044-8295.1993.tb02495.x

[4] Carpenter PJ Perceived control as a predictor of distress in children undergoing invasive medical procedures. J Pediatr Psychol 1992; 17:757–773 1484337 10.1093/jpepsy/17.6.757

[5] Newton P, Scambler S, Asimakopoulou K Marrying contraindications: Healthcare professionals perceptions of empowerment in the care of people with type 2 diabetes. Patient Educ Couns 2011; 85:e326–e329 21530141 10.1016/j.pec.2011.03.015

[6] D'Allessandro D, Dosa N Empowering children and families with information technology. Arch Pediatr Adolesc Med 2001;155:1131–1136 11576008 10.1001/archpedi.155.10.1131

[7] O'Brien CW, Moorey S Outlook and adaptation in advanced cancer: A systematic review. Psychooncology 2010; 12:1239–1249 20200856 10.1002/pon.1704

[8] Zeltzer LK, Recklitis C, Buchbinder D, et al. Psychological status in childhood cancer survivors: A report from the Childhood Cancer Survivor Study. J Clin Oncol 2009; 27:2396–2404 19255309 10.1200/JCO.2008.21.1433 PMC2677925

[9] Zegans L Stress and the development of somatic disorders. In: Goldberger L, Breznitz SE, eds. Handbook of Stress: Theoretical and Clinical Aspects. New York: Free Press; 1982, pp. 134–152

[10] Zebrack BJ, Zeltzer LK, Whitton J, et al. Psychological outcomes in long-term survivors of childhood leukemia, Hodgkin's disease, and non-Hodgkin's lymphoma: A report from the Childhood Cancer Survivor Study. Pediatrics 2002; 110:42–52 12093945 10.1542/peds.110.1.42

[11] Wicks L, Mitchell A The adolescent cancer experience: Loss of control and benefit finding. Eur J Cancer Care (Engl) 2010; 19:778–785 20088922 10.1111/j.1365-2354.2009.01139.x

[12] Carpenter PJ Perceived control as a predictor of distress in children undergoing invasive medical procedures. J Pediatr Psychol 1992; 17:757–773 1484337 10.1093/jpepsy/17.6.757

[13] Gariepy N, Howe N The therapeutic power of play: Examining the play of young children with leukaemia. Child Care Health Dev 2003; 29:523–537 14616910 10.1046/j.1365-2214.2003.00372.x

[14] Bradlyn A, Beale I, Kato P Psychoeducational interventions with pediatric cancer patients: Part I. Patient information and knowledge. J Child Fam Stud 2003; 12:257–277

[15] Beale I, Bradlyn A, Kato P Psychoeducational interventions with pediatric cancer patients: Part II. Effects of information and skills training on health-related outcomes. J Child Fam Stud 2003; 12:385–397

[16] Aujoulat I, d'Hoore W, Deccache A Patient empowerment in theory and practice: Polysemy or cacophony? Patient Educ Couns 2007; 66:13–20 17084059 10.1016/j.pec.2006.09.008

[17] Martiniuk M, Silva M, Amylon M, Barr R Camp programs for children with cancer and their families: Review of research progress over the past decade. Pediatr Blood Cancer 2014; 61:778–787 24395392 10.1002/pbc.24912

[18] Martiniuk AL, Amylon MD, Briery BG, et al. Camper learning and friendship at pediatric oncology camps in North America. J Psychosoc Oncol 2014; 32:234–244 24364990 10.1080/07347332.2013.874001

[19] Bluebond-Langner M, Perkel D, Goertzel T, et al. Children's knowledge of cancer and its treatment: Impact of an oncology camp experience. J Pediatr 1990; 116:207–213 2299490 10.1016/s0022-3476(05)82876-8

[20] Last BF, Stam H, Onland-van Nieuwenhuizen AM, Grootenhuis MA Positive effects of a psycho-educational group intervention for children with a chronic disease: First results. Patient Educ Couns 2007; 65:101–112 16870387 10.1016/j.pec.2006.06.017

[21] Scholten L, Willemen AM, Last BF, et al. Efficacy of psychosocial group intervention for children with chronic illness and their parents. Pediatrics 2013; 131:e1196–e1203 23478870 10.1542/peds.2012-2222

[22] Maurice-Stam H, Silberbusch LM, Last BF, Grootenhuis MA Evaluation of a psycho-educational group intervention for children treated for cancer: A descriptive pilot study. Psychooncology 2009; 18:762–766 19061197 10.1002/pon.1470

[23] Sadak KT, Connor C, DeLuca H Innovative educational approaches to engage and empower the adolescent and young adult childhood cancer survivor. Pediatr Blood Cancer 2013; 60:1919–1921 23956094 10.1002/pbc.24635

[24] Baumann FT, Bloch W, Beulertz J Clinical exercise interventions in pediatric oncology: A systematic review. Pediatr Res 2013; 74:366–374 23857296 10.1038/pr.2013.123

[25] Chang CW, Mu PF, Jou ST, et al. Systematic review and meta-analysis of nonpharmacological interventions for fatigue in children and adolescents with cancer. Worldviews Evid Based Nurs 2013; 10:208–217 23809656 10.1111/wvn.12007

[26] Kharrazi H Improving healthy behaviors in type 1 diabetic patients by interactive frameworks. AMIA Annu Symp Proc 2009; 2009:322–326 20351873 PMC2815464

[27] Howorka K, Pumprla J, Wagner-Nosiska D, et al. Empowering diabetes out-patients with structured education: Short-term and long-term effects of functional insulin treatment on perceived control over diabetes. J Psychosom Res 2000; 48:37–44 10750628 10.1016/s0022-3999(99)00074-4

[28] Lieberman DA Management of chronic pediatric diseases with interactive health games: Theory and research findings. J Ambul Care Manage 2001; 24:26–38 11189794 10.1097/00004479-200101000-00004

[29] Frame K Empowering preadolescents with ADHD: Demons or delights. ANS Adv Nurs Sci 2003, 26:131–139 12795541 10.1097/00012272-200304000-00005

[30] Wallston KA, Stein MJ, Smith CA Form C of the MHLC scales: A condition-specific measure of locus of control. J Pers Assess 1994; 63:534–553 7844739 10.1207/s15327752jpa6303_10

[31] Rogers ES, Chamberlin J, Ellison ML, Crean T A consumer-constructed scale to measure empowerment among users of mental health services. Psychiatr Serv 1997; 48:104201047 10.1176/ps.48.8.1042 9255837

[32] Wowra SA, McCarter R Validation of the empowerment scale with an outpatient mental health population. Psychiatr Serv 1999; 50:959–961 10402621 10.1176/ps.50.7.959

[33] Corrigan PW, Faber D, Rashid F, Leary M The construct validity of empowerment among consumers of mental health services. Schizphr Res 1999; 38:77–84 10.1016/s0920-9964(98)00180-7 10427613

[34] Gagnon M, Hibert R, Dube M, Dubois MF Development and validation of an instrument measuring individual empowerment in relation to personal health care: The Health Care Empowerment Questionnaire (HCEQ) Am J Health Promot 2006; 20:429–235 16871823 10.4278/0890-1171-20.6.429

[35] Kato PM, Cole SW, Bradlyn AS, Pollock BH A video game improves behavioral outcomes in adolescents and young adults with cancer: A randomized trial. Pediatrics 2008; 122:e305–e317 18676516 10.1542/peds.2007-3134

[36] Bakker L, Van Brakel WH Empowerment assessment tools in people with disabilities in developing countries. A systematic literature review. Lepr Rev 2012; 83:129–153 22997690

[37] Kuijpers W, Groen WG, Aaronson NK, van Haarten WH A systematic review of web-based interventions for patient empowerment and physical activity in chronic diseases: Relevance for cancer survivors. J Med Internet Res 2013;15:e37 23425685 10.2196/jmir.2281 PMC3636300

[38] van den Berg SW, Ploos van Amstel FJ, Ottevanger PO, et al. The Cancer Empowerment Questionnaire: Psychological empowerment in breast cancer survivors. J Psychosoc Oncol 2013; 31:565–583 24010533 10.1080/07347332.2013.825361

[39] Hara Y, Iwashita S, Okada A, et al. Development of a novel, short, self-completed questionnaire on empowerment for patients with type 2 diabetes mellitus and an analysis of factors affecting patient empowerment. Biopsychosoc Med 2014;8:19 25183994 10.1186/1751-0759-8-19 PMC4151376

[40] Maunsell E, Lauzier S, Brunet J, et al. Health-related empowerment in cancer: Validity of scales from the Health Education Impact Questionnaire. Cancer 2014; 120:3228–3236 24988944 10.1002/cncr.28847

[41] Johnson CE, Saunders CM, Phillips M, et al. Randomized controlled trial of shared care for patients with cancer involving general practitioners and cancer specialists. J Oncol Pract 2015 3 10 [Epub ahead of print]. doi: 10.1200/JOP.2014.001569 25758448

[42] Dragone MA, Bush PJ, Jones JK, et al. Development and evaluation of an interactive CD-ROM for children with leukemia and their families. Patient Educ Couns 2002; 46:97–307 10.1016/s0738-3991(01)00166-5 PMC3307387 11932129

[43] Price M, Yuen EK, Goetter EM, et al. mHealth: A mechanism to deliver more accessible, more effective mental health care. Clin Psychol Psychother 2014; 21:427–436 23918764 10.1002/cpp.1855 PMC3926903

[44] Jones JK, Kamani SA, Bush PJ, et al. Development and evaluation of an educational interactive CD-ROM for teens with cancer. Pediatr Blood Cancer 2010; 55:512–519 20533523 10.1002/pbc.22608 PMC3324939

[45] van Uden-Kraan CF, Drossaert CH, Taal E, et al. Participation in online patient support groups endorses patients' empowerment. Patient Educ Couns 2009; 74:1–69 10.1016/j.pec.2008.07.044 18778909

[46] Calear AL, Christensen H, Mackinnon A, et al. The YouthMood Project: A cluster randomized controlled trial of an online cognitive behavioral program with adolescents. J Consult Clin Psychol 2009; 77:1021–1032 19968379 10.1037/a0017391

[47] Das DA, Grimmer KA, Sparnon AL, et al. The efficacy of playing a virtual reality game in modulation in pain for children with acute burn injuries: A randomized controlled trial (ISRCTN8743556). BMC Pediatr 2005; 5:1–10 15745448 10.1186/1471-2431-5-1 PMC554986

[48] Bers MU, Gonzales-Heydrich J, DeMaso DR Use of a computer-based application in a pediatric hemodialysis unit: A pilot study. J Am Acad Child Adolesc Psychiatry 2003; 42:493–496 12649637 10.1097/01.CHI.0000046810.95464.68

[49] Bers MU, Beals LM, Chau C, et al. Use of a virtual community as a pyschosocial support system in pediatric transplantation. Pediatr Transplant 2010; 14:261–267 20470360 10.1111/j.1399-3046.2010.01271.x

[50] Li WH, Chung JO, Ho EK The effectiveness of therapeutic play, using virtual reality computer games, in promoting the psychological well-being of children hospitalised with cancer. J Clin Nurs 2011; 20:2135–2143 21651633 10.1111/j.1365-2702.2011.03733.x

[51] Beale IL, Kato PM, Marin-Bowling VM, et al. Improvement in cancer-related knowledge following use of a psychoeducational video game for adolescents and young adults with cancer. J Adolesc Health 2007; 41:263–270 17707296 10.1016/j.jadohealth.2007.04.006

[52] Graf DL, Pratt LV, Hester CN, Short KR Playing active video games increases energy expenditure in children. Pediatrics 2009; 124:534–540 19596737 10.1542/peds.2008-2851 PMC8329994

[53] Lanningham-Foster L, Jensen TB, Foster RC, et al. Energy expenditure of sedentary screen time compared with active screen time for children. Pediatrics 2006; 118:1831–1835 10.1542/peds.2006-1087 17142504

[54] Biddiss E, Irwin J Active video games to promote physical activity in children and youth: A systematic review. Arch Pediatr Adolesc Med 2010; 164:664–672 20603468 10.1001/archpediatrics.2010.104

[55] Esculier JF, Vaudrin J, Beriault P, et al. Home based balance training programme using Wii Fit with balance board for Parkinson's disease: A pilot study. J Rehabil Med 2012; 44:144–150 22266676 10.2340/16501977-0922

[56] Lange B, Flynn S, Proffitt R, et al. Development of an interactive game-based rehabilitation tool for dynamic balance training. Top Stroke Rehabil 2010; 17:345–352 21131259 10.1310/tsr1705-345

[57] Saposnic G, Teasell R, Mamdani M, et al. Effectiveness of virtual reality using Wii gaming technology in stroke rehabilitation: A pilot randomized clinical trial and proof of principle. Stroke 2010; 41:1477–1484 20508185 10.1161/STROKEAHA.110.584979 PMC4879973

[58] Russoniello CV, Fish M, O'Brien K The efficacy of casual videogame play in reducing clinical depression: A randomized controlled study. Games Health J 2013; 2:341–346 26197075 10.1089/g4h.2013.0010

[59] Fish MT, Russoniello CV, O'Brien K The efficacy of prescribed casual videogame play in reducing symptoms of anxiety: A randomized controlled study. Games Health J 2014; 3:291–297 26192483 10.1089/g4h.2013.0092

[60] Merry SN, Stasiak K, Shepherd M, et al. The effectiveness of SPARX, a computerised self help intervention for adolescents seeking help for depression: Randomised controlled non-inferiority trial. BMJ 2012; 344:e2598 22517917 10.1136/bmj.e2598 PMC3330131

[61] Redd WH, Jacobsen PB, Die-Trill M, et al. Cognitive/attentional distraction in the control of conditioned nausea in pediatric cancer patients receiving chemotherapy. J Consult Clin Psychol 1987; 55:391–395 3597954 10.1037//0022-006x.55.3.391

[62] Zeltzer LK, Dolgin MJ, LeBaron S, LeBaron C A randomized, controlled study of behavioral intervention for chemotherapy distress in children with cancer. Pediatrics 1991; 88:34–42 2057271

[63] Bruggers CS, Altizer R, Kessler R, et al. Incentive-based coupling of physical exercise with empowerment of fighting cancer in adolescent patients using a prototype interactive video game (PE game) [abstract 751272]. Presented at the 2012 Pediatric Academic Societies Annual Meeting, Boston, MA, April 28–May 1, 2012

[64] Caldwell C, Bruggers C, Altizer R, et al. The intersection of video games and patient empowerment: Case study of a real world application. In: Proceedings of the 9th Australasian Conference on Interactive Entertainment: Matters of Life and Death. IE'2013 ACM, New York, 2013: article 12. http://dx.doi.org/10.1145/2513002.2513018 or http://dl.acm.org/citation.cfm?doid=2513002.2513018 (accessed 64, 2015)

[65] Bruggers CS, Altizer RA, Kessler RR, et al. Patient-empowerment interactive technologies. Sci Transl Med 2012; 4:25–27 10.1126/scitranslmed.3004009 22993292

[66] Fiordelli M, Diviani N, Schulz PJ Mapping mHealth research: A decade of evolution. J Med Internet Res 2013; 15:e95 23697600 10.2196/jmir.2430 PMC3668610

[67] Adibi S Biomedical sensing analyzer (BSA) for mobile-health (mHealth)-LTE. IEEE J Biomed Health Inform 2014; 18:345–351 24403433 10.1109/JBHI.2013.2262076

[68] Kallander K, Tibenderana JK, Akpogheneta OJ, et al. Mobile health (mHealth) approaches and lessons for increased performance and retention of community health workers in low- and middle-income countries: A review. J Med Internet Res 2013; 15:e17 23353680 10.2196/jmir.2130 PMC3636306

[69] Brooke MJ, Thompson BM Food and Drug Administration regulation of diabetes-related mHealth technologies. J Diabetes Sci Technol 2013; 7:296–301 23566984 10.1177/193229681300700202 PMC3737627

[70] Price M, Yuen EK, Goetter EM, et al. mHealth: A mechanism to deliver more accessible, more effective mental health care. Clin Psychol Psychother 2014; 21:427–436 23918764 10.1002/cpp.1855 PMC3926903

[71] Chomutare T, Tatara N, Arsand E, Hartvigsen G Designing a diabetes mobile application with social network support. Stud Health Technol Inform 2013; 188:58–64 23823289

[72] Quinn CC, Clough SS, Minor JM, et al. WellDoc mobile diabetes management randomized controlled trial: Change in clinical and behavioral outcomes and patient and physician satisfaction. Diabetes Technol Ther 2008; 10:160–168 18473689 10.1089/dia.2008.0283

[73] Peeples MM, Iyer AK, Cohen JL Integration of a mobile-integrated therapy with electronic health records: Lessons learned. J Diabetes Sci Technol 2013; 7:602–611 23759392 10.1177/193229681300700304 PMC3869127

[74] Dennis TA, O'Toole LJ Mental health on the go: Effects of a gamified attention bias modification mobile application in trait anxious adults. Clin Psychol Sci 2014; 2:576–590 26029490 10.1177/2167702614522228 PMC4447237

[75] Gaggliol A, Cipresso P, Serino S, et al. Positive technology: A free mobile platform for the self-management of psychological stress. Stud Health Technol Inform 2014; 199:25–29 24875684

[76] Shin IH, Cha J, Cheon GW et al. Automatic stress-relieving music recommendation system based on photoplethysmography-derived heart rate variability analysis. Conf Proc IEEE Eng Med Biol Soc 2014; 2014:6402–6405 25571461 10.1109/EMBC.2014.6945093

[77] Wiederhold BK, Boyd C, Sulea C, Riva G Marketing analysis of a positive technology app for the self-management of psychological stress. Stud Health Technol Inform 2014; 199:83–87 24875696

[78] Hayes DF, Markus HS, Leslie RD, Topol EJ Personalized medicine: Risk prediction, targeted therapies, and mobile health technology. BMC Med 2014; 12:37 24580858 10.1186/1741-7015-12-37 PMC3938085

[79] Charney DS Psychobiological mechanisms of resilience and vulnerability: Implications for successful adaptation to extreme stress. Am J Psychiatry 2004; 161:195–216 14754765 10.1176/appi.ajp.161.2.195

[80] Southwick SM, Charney DS The science of resilience: Implications for the prevention and treatment of depression. Science 2012; 338:79–82 23042887 10.1126/science.1222942

[81] Nestler EJ, Carlezon WA Jr. The mesolimbic dopamine reward circuit in depression. Biol Psychiatry 2006; 59:1151–1159 16566899 10.1016/j.biopsych.2005.09.018

[82] Feder A, Nestler EJ, Charney DS Psychobiology and molecular genetics of resilience. Nat Rev Neurosci 2009; 10:446–457 19455174 10.1038/nrn2649 PMC2833107

[83] Wu G, Feder A, Cohen H, et al. Understanding resilience. Front Behav Neurosci 2013; 7:10–25 23422934 10.3389/fnbeh.2013.00010 PMC3573269

[84] Shin LM, Liberzon I The neurocircuitry of fear, stress, and anxiety disorders. Neuropsychopharmacology 2010; 35:169–191 19625997 10.1038/npp.2009.83 PMC3055419

[85] Curtis WJ, Cicchetti D Moving research on resilience into the 21st century: Theoretical and methodological considerations in examining the biological contributors to resilience. Dev Psychopathol 2003; 15:773–810 14582940 10.1017/s0954579403000373

[86] Vaisvaser S, Lin T, Admon R, et al. Neural traces of stress: Cortisol related sustained enhancement of amygdala-hippocampal functional connectivity. Front Hum Neurosci 2013; 7:313–324 23847492 10.3389/fnhum.2013.00313 PMC3701866

[87] Wolf OT The influence of stress hormones on emotional memory: Relevance for psychopathology. Acta Psychol (Amst) 2008; 127:513–531 17900515 10.1016/j.actpsy.2007.08.002

[88] Thorsell A Brain neuropeptide Y and corticotropin-releasing hormone in mediating stress and anxiety. Exp Biol Med 2010; 235:1163–1167 10.1258/ebm.2010.009331 20881320

[89] Sajdyk TJ, Shekhar A, Gehlert DR Interactions between NPY and CRF in the amygdala to regulate emotionality. Neuropeptides 2004; 38:225–234 15337374 10.1016/j.npep.2004.05.006

[90] McEwen BS Physiology and neurobiology of stress and adaptation: Central role of the brain. Physiol Rev 2007; 87:873–904 17615391 10.1152/physrev.00041.2006

[91] Southwick SM, Vythilingam M, Charney DS The psychobiology of depression and resilience to stress: Implications for prevention and treatment. Annu Rev Clin Psychol 2005; 1:255–291 17716089 10.1146/annurev.clinpsy.1.102803.143948

[92] O'Doherty JP Reward representations and reward-related learning in the human brain: Insights from neuroimaging. Curr Opin Neurobiol 2004; 14:769–776 15582382 10.1016/j.conb.2004.10.016

[93] Price JL, Drevets WC Neurocircuitry of mood disorders. Neuropsychopharmacology 2010; 35:192–216 19693001 10.1038/npp.2009.104 PMC3055427

[94] Kask A, Harro J, von Horsten S, et al. The neurocircuitry and receptor subtypes mediating anxiolytic-like effects of neuropeptide Y. Neurosci Biobehav Rev 2002; 26:259–283 12034130 10.1016/s0149-7634(01)00066-5

[95] Heilig M The NPY system in stress, anxiety and depression. Neuropeptides 2004; 38:213–224 15337373 10.1016/j.npep.2004.05.002

[96] Morgan CA, Wang S, Rasmusson A, et al. Relationship among plasma cortisol, catecholamines, neuropeptide Y, and human performance during exposure to uncontrollable stress. Psychosom Med 2001; 63:412–422 11382268 10.1097/00006842-200105000-00010

[97] Cole SW, Yoo DJ, Knutson B Interactivity and reward-related neural activation during a serious videogame. PLoS One 2012; 7:e33909 22442733 10.1371/journal.pone.0033909 PMC3307771

[98] Vythilingam M, Nelson EE, Scaramozza M, et al. Reward circuitry in resilience to severe trauma: An fMRI investigation of resilient Special Forces soldiers. Psychiatry Res 2009; 172:75–77 19243926 10.1016/j.pscychresns.2008.06.008 PMC2760852

[99] Petros N, Opacka-Juffry J, Huber JH Psychometric and neurobiological assessment of resilience in a non-clinical sample of adults. Psychoneuroendocrinology 2013; 38:2099–2108 23642338 10.1016/j.psyneuen.2013.03.022

[100] Goodyear IM, Park J, Netherton CM, Herbert J Possible role of cortisol and dehydroepiandrosterone in human development and psychopathology. Br J Psychiatry 2001; 179:243–249 11532802 10.1192/bjp.179.3.243

[101] Shin LM, Davis FC, Vanelzakker MB, et al. Neuroimaging predictors of treatment response in anxiety disorders. Biol Mood Anxiety Disord 2013; 3:1591 10.1186/2045-5380-3-15 PMC3750275 23915782

[102] Darviri C, Alexopoulos EC, Artemiadis A, et al. The Healthy Lifestyle and Personal Control Questionnaire (HLPCQ): A novel tool for assessing self-empowerment thorough a constellation of daily activities. BMC Public Health 2014; 14:995–105 25253039 10.1186/1471-2458-14-995 PMC4192765

[103] Hara Y, Iwashita S, Okada A, et al. Development of a novel short, self-completed questionnaire on empowerment for patients with type 2 diabetes mellitus and an analysis of factors affecting patient empowerment. Biopsychosoc Med 2014; 8:19–31 25183994 10.1186/1751-0759-8-19 PMC4151376

[104] Bulaj G Combining non-pharmacological treatments with pharmacotherapies for neurologic disorders: A unique interface of the brain, drug-device, and intellectual property. Front Neurol 2014; 5:1–7 25071711 10.3389/fneur.2014.00126 PMC4095562

[105] O'Doherty JP Reward representations and reward-related learning in the human brain: Insights from neuroimaging. Curr Opin Neurobiol 2004; 14:769–776 15582382 10.1016/j.conb.2004.10.016

[106] Baranowski T, Buday R, Thompson BI, Baranowski J Playing for real: Video games and stories for health related behavior change. Am J Prev Med 2008; 34:74–82.e10 18083454 10.1016/j.amepre.2007.09.027 PMC2189579

[107] Brown-Johnson CG, Berrean B, Cataldo JK Development and usability evaluation of the mHealth tool for lung cancer (mHealth TLC): A virtual word health game for lung cancer patients. Patient Educ Couns 2015; 98:506–511 25620075 10.1016/j.pec.2014.12.006 PMC4451946

